# Exerting an influence on evolution

**DOI:** 10.7554/eLife.55952

**Published:** 2020-03-25

**Authors:** Charles C Roseman

**Affiliations:** Department of Evolution, Ecology, and Behavior, School of Integrative BiologyUniversity of Illinois, Urbana-ChampaignUrbanaUnited States

**Keywords:** evo-devo, rodent, molar, line of least resistance, developmental constraint, developmental variability, Mouse

## Abstract

Experiments on mice have shown that developmental processes are influencing the generation of phenotypic variation in a way that shapes evolution.

**Related research article** Hayden L, Lochovska K, Sémon M, Renaud S, Delignette-Muller ML, Vilcot M, Peterkova R, Hovorakova M, Pantalacci S. 2020. Developmental variability channels mouse molar evolution. *eLife* **9**:e50103. doi: 10.7554/eLife.50103

Variation presents us with a conundrum in evolutionary biology. On one hand, natural selection requires the presence of heritable variation in phenotypes to effect evolutionary change in a population. On the other, the variation we see in a given population today is not the variation that allowed this population to evolve to its present state. Rather, this original variation has been radically altered by selection and random genetic drift during the course of evolution. As such, understanding the ways in which new heritable variation is generated in populations via developmental processes is a key problem in evolutionary biology (Cheverud, 1984; Hallgrímsson et al., 2009).

Several studies that compare the morphology of certain teeth in different species of mice present tantalizing evidence that the first molars in the upper jaw get longer by speeding up the growth of their front-most part during development (Renaud et al., 2011; Renaud et al., 2018; Ledevin et al., 2016). This often results in the addition of an extra cusp to the front of the upper first molar. This means that development might specify a 'line of least evolutionary resistance' along which a population is biased to evolve.

Now, in eLife, Sophie Pantalacci (Université de Lyon), Maria Hovorakova (Czech Academy of Sciences) and co-workers – including Luke Hayden as first author – report the results of experiments in which they tracked the activity of signaling centers in two strains of mice (called DUHi and FVB) during development (Hayden et al., 2020). This allowed them to test a series of hypotheses about what causes variations in the length of the upper first molar.

Tooth development is well characterized in mammals. Early on, signaling centers corresponding to enamel knots are laid down in the epithelial precursors of the molar row (Jernvall et al., 1994; Kavanagh et al., 2007; Sadier et al., 2019). These centers then anchor the later development of the tooth crown and, eventually, the cusps of the teeth. Complex interactions among spatially and temporally varying developmental factors guide the transformation of these early tooth rudiments into adult teeth. Less well understood is how modifying these processes may result in variable phenotypic outcomes.

Previous studies have provided empirical and theoretical evidence for developmental biases on tooth morphology. However, few of these studies have bridged the gap between morphology in embryos and adults as comprehensively as the latest work, and few have directly addressed the developmental underpinnings of an evolutionary change that appears to occur repeatedly in nature. Hayden et al. began their investigation by demonstrating that the differences between the two strains they chose indeed mirror morphological transitions among wild mouse populations. In this case, the DUHi mice have larger first molars, often with an extra cusp (Figure 1A).

**Figure 1.**
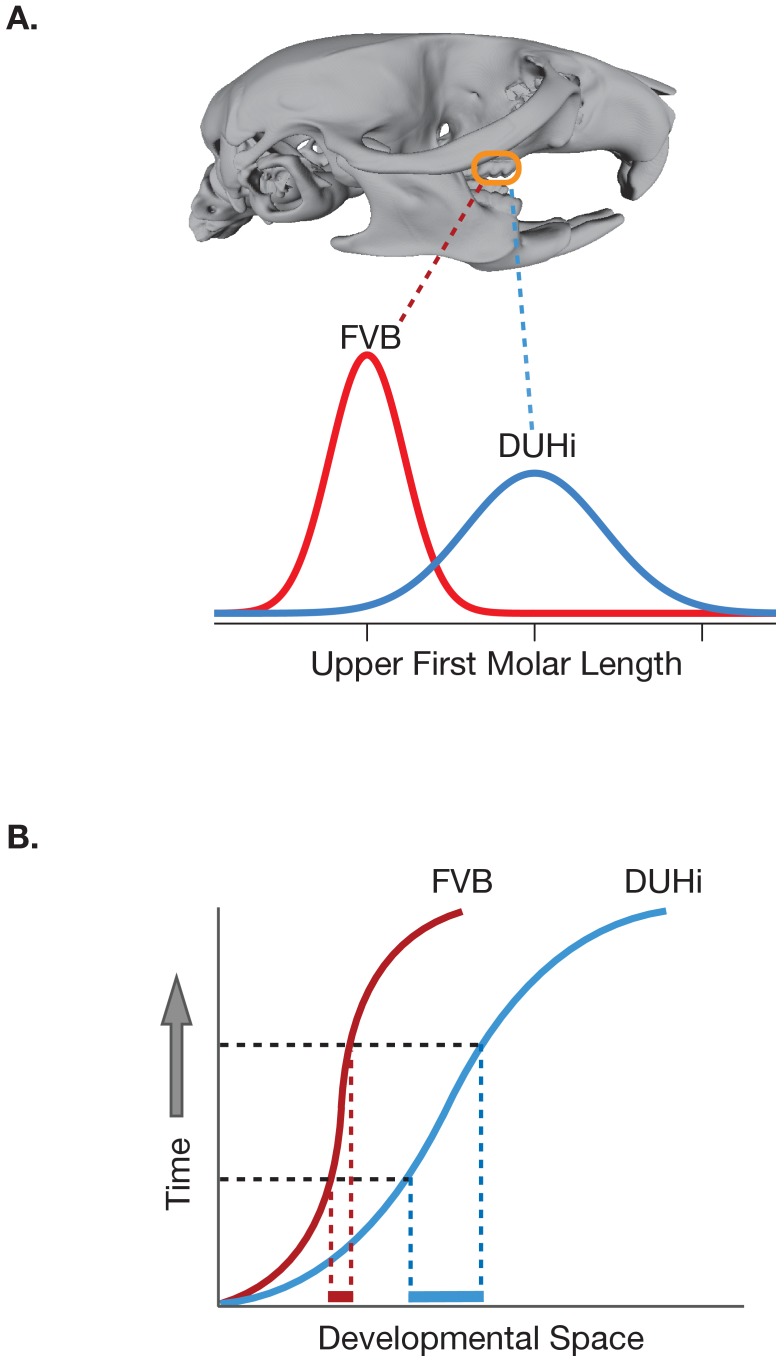
Differences in the developmental trajectories of two strains of mice result in different degrees of phenotypic variation. (**A**) The upper first molar is longer in DUHi mice (blue line) than in FVB mice (red line); the variance in the length is also higher in the DUHi mice. (**B**) This difference in variance arises from the fact that the developmental trajectory of the DUHi mouse (curved blue line) occupies more of the developmental space (solid blue line along the x-axis) in any interval of time (defined by the dashed black lines) than in the FVB mice (solid red line). Mouse skull image provided by Benedikt Hallgrímsson and Rebecca Green.

The researchers then modeled the transition from early tooth precursor to full adult tooth by tracing tooth development at different ages in a 'developmental space' that expresses the possible morphological and gene expression states that developing teeth could occupy (Figure 1B). Comparing the developmental trajectories of the two species revealed that they are on distinct developmental paths. One notable difference is that a signaling center hypothesized to be a rudiment of a premolar that has been lost during rodent evolution persists into later developmental stages in the DUHi mice. Further, differences in the degree to which several sets of core dental genes were expressed were observed during key parts of development.

A particularly interesting result is that one of the strains (DUHi) occupies more varied positions in the developmental space at any given actual age stage (Figure 1B). This suggests the presence of strong differences between the two strains in terms of how development integrates variable influences and turns them into morphological differences in adult teeth. This added developmental variation appears to translate into more variance in the length of the first molar in the DUHi strain (Figure 1A).

Several questions remain. The strains compared in Hayden et al. are inbred, which means that they have no genetic variation within groups. This raises the possibility of a multitude of different ways in which the same developmental changes might be accomplished from a genetic perspective. Similar phenotypic outcomes can result from very different combinations of developmental events. Future work on more variable populations of mice would help resolve this issue. Moreover, the role of natural selection itself in causing dental differences between populations of mice is yet to be described. The hypothesis of a strong developmental bias in molar variation proposed by Hayden et al. is an ideal way to structure future investigations into the evolution of dental diversity.
